# CXCR4- and CCR5-Tropic HIV-1 Clones Are Both Tractable to Grow in Rhesus Macaques

**DOI:** 10.3389/fmicb.2018.02510

**Published:** 2018-10-18

**Authors:** Naoya Doi, Tomoyuki Miura, Hiromi Mori, Hiromi Sakawaki, Takaaki Koma, Akio Adachi, Masako Nomaguchi

**Affiliations:** ^1^Department of Microbiology, Graduate School of Medical Sciences, Tokushima University, Tokushima, Japan; ^2^Laboratory of Primate Model, Institute for Frontier Life and Medical Sciences, Kyoto University, Kyoto, Japan; ^3^Non-human Primate Experimental Facility, Institute for Frontier Life and Medical Sciences, Kyoto University, Kyoto, Japan; ^4^Department of Microbiology, Kansai Medical University, Hirakata, Japan

**Keywords:** HIV-1, primate model, rhesus macaque, HIV-1rmt, CXCR4-tropic, CCR5-tropic

## Abstract

A major issue for present HIV-1 research is to establish model systems that reflect or mimic viral replication and pathogenesis actually observed in infected humans. To this end, various strategies using macaques as infection targets have long been pursued. In particular, experimental infections of rhesus macaques by HIV-1 derivatives have been believed to be best suited, if practicable, for studies on interaction of HIV-1 and humans under various circumstances. Recently, through *in vitro* genetic manipulations and viral cell-adaptations, we have successfully generated a series of HIV-1 derivatives with CXCR4-tropism or CCR5-tropism that grow in macaque cells to various degrees. Of these viruses, those with best replicative potentials can grow comparably with a pathogenic SIVmac in macaque cells by counteracting major restriction factors TRIM5, APOBEC3, and tetherin proteins. In this study, rhesus macaques were challenged with CXCR4-tropic (MN4/LSDQgtu) or CCR5-tropic (gtu + A4CI1) virus. The two viruses were found to productively infect rhesus macaques, being rhesus macaque-tropic HIV-1 (HIV-1rmt). However, plasma viral RNA was reduced to be an undetectable level in infected macaques at 5–6 weeks post-infection and thereafter. While replicated similarly well in rhesus peripheral blood mononuclear cells, MN4/LSDQgtu grew much better than gtu + A4CI1 in the animals. To the best of our knowledge, this is the first report demonstrating that HIV-1 derivatives (variants) grow in rhesus macaques. These viruses certainly constitute firm bases for generating HIV-1rmt clones pathogenic for rhesus monkeys, albeit they grow more poorly than pathogenic SIVmac and SHIV clones reported to date.

## Introduction

HIV-1 has emerged from ancestral viruses by extensive recombination and/or adaptation events (Sharp and Hahn, 2011). It exhibits an exquisitely complicated replication format, about which much remains to be precisely clarified (Blanco-Melo et al., 2012; Engelman and Cherepanov, 2012; Grütter and Luban, 2012; Malim and Bieniasz, 2012; Freed and Martin, 2013; Campbell and Hope, 2015; Dahabieh et al., 2015; Freed, 2015; Nakayama and Shioda, 2015; Harada and Yoshimura, 2017; Heusinger and Kirchhoff, 2017; Yamashita and Engelman, 2017; Foster et al., 2018). HIV-1 is highly adapted to humans in nature, and thus strictly tropic only for humans and chimpanzees. Following infection, HIV-1 persists in humans, and after lengthy persistent state, ultimately causes AIDS-related diseases and AIDS in most cases if not treated appropriately. Due to the exceptionally narrow host range of HIV-1, primarily based on its sophisticatedly regulated replication, animal models for experimental infections have been difficult to develop from the dawn period to the present stage of HIV-1 research. Ambitious attempts to establish *in vivo* systems effective and potent for model studies on HIV-1 continue to be one of major approaches toward basic and clinical studies on HIV-1 and AIDS.

A number of animal models have been proposed and tested so far to perform *in vivo* studies on HIV-1 (Ambrose et al., 2007; Nomaguchi et al., 2008, 2011; Hatziioannou and Evans, 2012; Nishimura and Martin, 2017). Surrogate models for HIV-1 infection in humans include feline immunodeficiency virus (FIV) in cats and simian immunodeficiency virus isolated from the rhesus macaque (SIVmac) infection in macaques (Hirsch et al., 1989; Slee et al., 1995; Veazey et al., 1998; Auwerx et al., 2004; Sparger, 2006; Ambrose et al., 2007; Chahroudi et al., 2012; Hatziioannou and Evans, 2012). Of note, the SIVmac/macaque system has been frequently and widely used for various study purposes, but not the FIV/cat model (Hatziioannou and Evans, 2012). As for the other animal models, because none of experimental animals are susceptible to HIV-1 infection as described above, challenge viruses and/or host animals need to be artificially manipulated. Small animal model systems, i.e., transgenic mice/HIV-1, transgenic rats/HIV-1, and transgenic rabbits/HIV-1, have been unsuccessful due to the lack of robust HIV-1 replication and/or disease progression (Dunn et al., 1995; Browning et al., 1997; Keppler et al., 2002; Hatziioannou and Evans, 2012). Exception is the humanized mouse model (human immune system mouse model). A number of humanized mouse models have been generated and extensively used for HIV-1 research (Hatziioannou and Evans, 2012). However, there is clearly an unavoidable limitation for the humanized mouse system. Since humanized mice cannot have a complete functional human immune system (cellular and humoral acquired immunity), they cannot reproduce typical features of HIV-1 replication and pathogenesis *in vivo* in response to HIV-1 infection. As an alternative for SIVmac in the macaque system, a chimeric virus designated SHIV has been extensively and successfully utilized for input virus for experimental infection of macaques since its initial description by us (Shibata et al., 1991; Sakuragi et al., 1992; Shibata and Adachi, 1992). SHIVs are genetically engineered virus clones that basically carry the *env* (Shibata et al., 1991), *pol*-reverse transcriptase (RT) (Uberla et al., 1995), or *pol*-protease (Ishimatsu et al., 2007) gene of HIV-1 in the backbone of SIVmac genome. An SHIV carrying HIV-1 *pol*-RT and Env also has been constructed (Smith et al., 2010). Of these SHIVs, Env-SHIV has been most widely and successfully used, especially for immunotherapy research for HIV-1 (Nishimura and Martin, 2017).

## Macaque-Tropic HIV-1 Derivative Clones

To experimentally and demonstratively perform studies on HIV-1 replication and pathogenesis in the presence of host innate and acquired immunity, non-human primate (NHP) models are essentially required. For this aim, three species of macaques, rhesus, cynomolgus, and pig-tailed macaques have been currently used for HIV-1 infection experiments. Macaques belong to Old World monkeys, and are susceptible to SIVmac but not to HIV-1 (Nakayama and Shioda, 2012). Nevertheless, rhesus macaques of Indian origin are best characterized, most utilized and most successfully used NHPs for SIV- or SHIV-based model studies on HIV-1/AIDS (Nomaguchi et al., 2011; Hatziioannou and Evans, 2012). Although pig-tailed macaques have been quite frequently and widely used for HIV-1 model studies, they unusually rapidly progress to AIDS upon infection with SIVmac (Batten et al., 2006; Hatziioannou and Evans, 2012; Klatt et al., 2012). In addition, they lack the TRIM-mediated restriction against HIV-1 Gag-capsid (CA), and their immunological background is not so well characterized relative to that of rhesus macaques (Igarashi et al., 2007; Hatziioannou et al., 2009; Hatziioannou and Evans, 2012; Nakayama and Shioda, 2012). Cynomolgus macaques have not been so widely used for HIV-1/AIDS model studies (Hatziioannou and Evans, 2012). SIVmac and SHIV are found to be less pathogenic to cynomolgus monkeys, and their immunological features are also less characterized (Reimann et al., 2005; Pendley et al., 2008; Budde et al., 2010; Dietrich et al., 2011; Hatziioannou and Evans, 2012).

Although SIV and SHIV infect rhesus macaques and cause AIDS in the animals, their genomes are very different from HIV-1 genome. In addition to profound sequence variations, their genome compositions are distinct. *Vpx* gene is not present in HIV-1 genome, whereas *vpu* gene does not exist in SIVmac genome. SHIVs are SIVmac-derivative chimeric viruses as described above. Therefore, it has been consensus to have macaque-tropic HIV-1 derivative clones for experimental macaque infections. To overcome the species barrier against HIV-1, and generate the HIV-1 infection model system using macaques, it was absolutely necessary to pinpoint the viral genomic regions responsible for the narrow host range of HIV-1. We and others have independently and almost simultaneously identified the regions, i.e., Gag-CA and Vif, and successfully generated macaque cell-tropic HIV-1 clones [designated simian-tropic (st) HIV-1, HIV-1 derivative, or HIV-1mt (macaque-tropic)] (Hatziioannou et al., 2006; Kamada et al., 2006; Nomaguchi et al., 2011; Saito et al., 2011).

**FIGURE 1:**
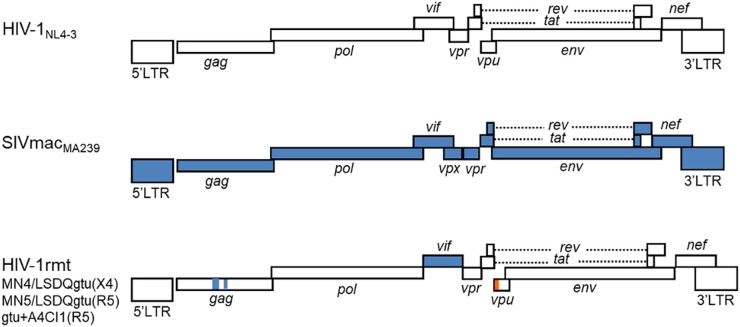
Basic genome structure of HIV-1rmt clones. The three HIV-1rmt clones indicated have been constructed from three distinct primate immunodeficiency viruses as shown. Genomic regions of HIV-1rmt clones derived from HIV-1 NL4-3, SIVmac MA239, and SIVgsn 166 (SIV isolated from the greater spot-nosed monkey) are depicted by white, blue, and orange areas, respectively. Generation and characterization of HIV-1 NL4-3 (Adachi et al., 1986), SIVmac MA239 (Shibata et al., 1991), CXCR4-tropic HIV-1rmt designated MN4/LSDQgtu (Nomaguchi et al., 2013b; Nomaguchi et al., 2017), CCR5-tropic HIV-1rmt designated MN5/LSDQgtu (Nomaguchi et al., 2013b; Nomaguchi et al., 2017), and CCR5-tropic HIV-1rmt designated gtu + A4CI1 (Doi et al., 2017) have been fully described previously. MN4/LSDQgtu and MN5/LSDQgtu carry growth-enhancing mutations in Gag-capsid, Pol-integrase, and Env regions as previously described (Nomaguchi et al., 2013a,b). GenBank accession numbers for sequences of NL4-3, MA239, 166, MN4/LSDQgtu, and MN5/LSDQgtu are AF324493, M33262, AF468659, LC315178, and LC315179, respectively.

From the prototype HIV-1mt designated NL-DT5R (Kamada et al., 2006), we have modified its genome stepwise and improved its replication potential in macaque cells through sequentially introducing necessary mutations/variations by *in vitro* mutagenesis coupled with viral genome adaptation in cells (Nomaguchi et al., 2008, 2011, 2013a,b,c; Kuroishi et al., 2009; Saito et al., 2011; Doi et al., 2017). Of particular note and importance, we resultantly have obtained CXCR4-tropic and CCR5-tropic HIV-1rmt (rhesus macaque-tropic) clones that grow well in rhesus cells (Nomaguchi et al., 2013b, 2014; Doi et al., 2017). **Figure 1** shows the basic genome structure of our HIV-1rmt clones. CXCR4-tropic MN4/LSDQgtu is demonstrated to be resistant to major anti-restriction factors (TRIM5α, APOBEC3, and tetherin) present in cynomolgus and rhesus macaque cells (Nomaguchi et al., 2013b), and grows best in rhesus cells among macaque-tropic HIV-1 derivative clones to the best of our knowledge (Nomaguchi et al., 2014).

## Growth Of HIV-1_RMT_ Clones in Rhesus Macaques

**FIGURE 2:**
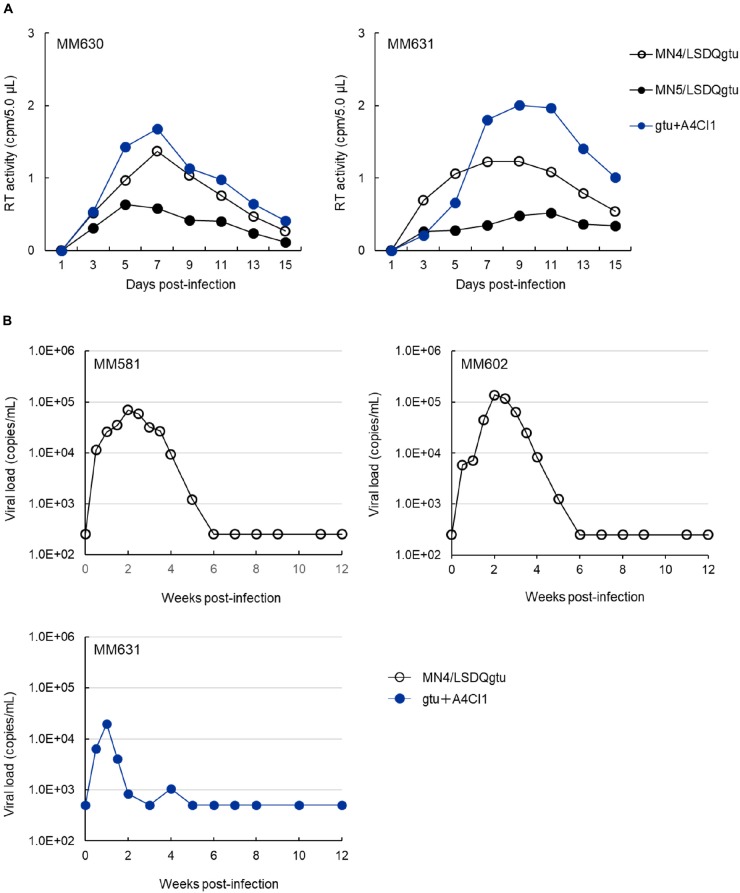
Growth property of HIV-1rmt clones in rhesus PBMCs and individuals. **(A)** Viral replication kinetics in rhesus PBMCs infected with CXCR4-tropic MN4/LSDQgtu, CCR5-tropic MN5/LSDQgtu, or CCR5-tropic gtu + A4CI1. PBMCs were prepared from rhesus macaques MM630 and MM631, and spin-infected with cell-free viruses obtained from transfected 293T cells as previously described (Nomaguchi et al., 2013b, 2014). Cell numbers and input viral amounts used were 2.0 × 10^6^ and 4.1 × 10^6^ RT units, respectively. **(B)** Kinetics of plasma viral loads in rhesus macaques inoculated with CXCR4-tropic MN4/LSDQgtu or CCR5-tropic gtu + A4CI1. Rhesus macaques MM581, MM602, and MM631 were infected with cell-free viruses obtained from transfected 293T cells, and monitored for viral RNAs in plasma as previously described (Otsuki et al., 2014; Ishida et al., 2016). MM581 and MM602 were inoculated intravenously with 4.3 × 10^5^ TCID_50_ of MN4/LSDQgtu as determined in a macaque cell line HSC-F (Akari et al., 1999). MM631 was inoculated with gtu + A4CI1 intravenously (5.0 × 10^6^ TCID_50_ in HSC-F cells) and intraperitoneally (1.5 × 10^7^ TCID_50_ in HSC-F cells). Infection experiments (MM581/MM602 and MM631) were separately and independently conducted, and the detection limits for the MM581/MM602 and MM631 experiments were 250 and 500 copies/ml, respectively. The *TRIM5* genotypes as analyzed by the previously described method (Wilson et al., 2008) for MM581, MM602, MM630, and MM631 are *TRIM5^TFP/TFP^* (*Mamu-1/Mamu-3*), *TRIM5^TFP/TFP^* (*Mamu-3/Mamu-3*), *TRIM5^TFP/TFP^* (*Mamu-3/Mamu-3*), and *TRIM5^Q/Q^* (*Mamu-4/Mamu-4*), respectively.

For routine check and characterization of our HIV-1rmt clones, an immortalized lymphocyte cell line of rhesus origin was established, and designated M1.3S (Doi et al., 2010, 2011). Generally, CCR5-tropic MN5/LSDQgtu grew more poorly in M1.3S cells and rhesus peripheral blood mononuclear cells (PBMCs) than CXCR4-tropic MN4/LSDQgtu (Doi et al., 2013; our unpublished data). After confirming the replication ability in M1.3S cells, we comparatively examined the three HIV-1rmt clones (MN4/LSDQgtu, MN5/LSDQgtu, and gtu + A4CI1 in **Figure 1**) for their growth properties in rhesus PBMCs as previously described (Nomaguchi et al., 2013b, 2014). Representative results are shown in **Figure 2A**. As is clear, MN4/LSDQgtu grew much better than MN5/LSDQgtu. However, notably, gtu + A4CI1 that carries an *env* gene from a clinical HIV-1 isolate, and had been adapted in M1.3S cells (Doi et al., 2017), grew comparably well with MN4/LSDQgtu in two preparations of rhesus PBMCs. The results in **Figure 2A** clearly show that the three HIV-1 derivative clones are tropic for rhesus PBMCs, and that CCR5-tropic gtu + A4CI1 grow much better than CCR5-tropic MN5/LSDQgtu in the cells. We then inoculated MN4/LSDQgtu and gtu + A4CI1 into rhesus macaques to determine whether the two virus clones can replicate in the animals to a readily detectable level, i.e., to confirm that they are really rhesus macaque-tropic. As shown in **Figure 2B**, both viruses significantly grew in the animals as monitored by viral RNA in plasma, a definite experimental indication as HIV-1rmt. However, virus production reached the peak (∼10^5^ viral RNA copies/ml for MN4/LSDQgtu and ∼10^4^ viral RNA copies/ml for gtu + A4CI1) at 1–2 weeks post-inoculation and was transient, being undetectable at 5–6 weeks post-inoculation. Numbers of circulating CD4-positive T-lymphocytes in the three animals were not affected significantly by the infections (data not shown). While the peak level was 10- to 10^3^-fold lower than that for pathogenic SIVmac and SHIV, MN4/LSDQgtu grew obviously better than gtu + A4CI1 (compare the results for MM581/602 and MM631). In another series of infection experiments using two rhesus macaques, where multiple CCR5-tropic virus clones (gtu + A4CI1 plus a few of the other distinct CCR5-tropic clones) were simultaneously inoculated into the animals to anticipate growth-enhancing adaptive recombination/mutations to occur, both monkeys were certainly infected with the virus(es), and transiently produced viral RNAs in plasma as observed for infected MM631 in **Figure 2B** (data not shown). It has been well established that most rhesus *TRIM5* alleles (*Mamu-1* to *Mamu-6*) affect HIV-1 replication, but not *Mamu-7*. While rhesus TRIM5α proteins encoded by *TRIM5^TFP^* (*Mamu-1* to *Mamu-3*) and *TRIM5^Q^* (*Mamu-4* to *Mamu-6*) inhibit HIV-1 replication (TFP protein is more potent than Q protein), rhesus TRIM5CypA encoded by *TRIM5^CypA^* (*Mamu-7*) does not influence HIV-1 replication (Price et al., 2009; Ylinen et al., 2010; Nakayama and Shioda, 2012, 2015; Nomaguchi et al., 2013b). We therefore determined the *TRIM5* genotype of the four rhesus macaques used in **Figure 2** as previously described (Wilson et al., 2008; Nomaguchi et al., 2013b). Macaques MM581, MM602, MM630, and MM631 were found to carry *TRIM5^TFP/TFP^* (*Mamu-1/Mamu-3*), *TRIM5^TFP/TFP^* (*Mamu-3/Mamu-3*), *TRIM5^TFP/TFP^* (*Mamu-3/Mamu-3*), and *TRIM5^Q/Q^* (*Mamu-4/Mamu-4*), respectively. Based on these results, distinct growth efficiencies of MN4/LSDQgtu and gtu + A4CI1 observed in infected MM581/MM602 and MM631 (MN4/LSDQ grew much better than gtu + A4CI1 in rhesus macaques) are unlikely to be attributable to the *TRIM5* alleles. In summary, we have demonstrated here that both CXCR4-tropic and CCR5-tropic HIV-1rmt clones readily infected rhesus macaques with restrictive *TRIM5* alleles, albeit less efficiently relative to standard SIV/SHIVs pathogenic for rhesus macaques.

## Concluding Remarks

This is the first report to demonstrate the capability of CXCR4-tropic and CCR5-tropic HIV-1 derivative viruses to grow in rhesus macaques. Thus far, pig-tailed and cynomolgus macaques have been the only NHPs to perform *in vivo* infection studies on HIV-1/AIDS using viruses genetically recognizable as HIV-1 (Igarashi et al., 2007; Hatziioannou et al., 2009, 2014; Saito et al., 2011, 2013; Thippeshappa et al., 2011; Otsuki et al., 2014; Peng et al., 2018). The rhesus macaque is by far the best suited NHP species for HIV-1 model studies from various points of views as described above. Our results described here would forward numerous research projects in this research field. Considering that MN4/LSDQgtu does grow considerably in rhesus macaques, it can be utilized to analyze the early infection stage of HIV-1 *in vivo*. Also, functional roles for HIV-1 accessory proteins in viral replication *in vivo*, which remains to be elucidated, could be examined by mutational analyses on MN4/LSDQgtu. Needless to mention, various basic and clinical projects become practicable when pathogenic HIV-1rmt clones are available.

Issues to be addressed in the near future can be summarized as follows. (i) Obviously, to increase heterologous viral populations after infection, improving the replication capability of the present HIV-1rmt clones is required. For viral persistence in individuals, viral variations to certain extent may be essential. In this regard, our experience indicates that Gag-Pol region is still amendable by *in vitro* mutagenesis. Better-growing CCR5-tropic viruses are particularly necessary to mimic the HIV-1’s natural infection course in individuals. (ii) More sequences derived from distinct clinical isolates may be needed to generate new HIV-1rmt clones. This attempt may result in obtaining new useful HIV-1rmt variants. (iii) Viral adaptation in rhesus macaques, in addition to the adaptation in cell cultures, should be considered to obtain virus clones pathogenic for rhesus macaques. Finally, in conclusion, studies in these directions are in progress in our laboratories.

## Animal Experiments

Monkey experiments in this study were carried out in biosafety level 3 animal facilities, in compliance with the institutional regulations approved by the Committee for Experimental Use of Non-human Primates of the Institute for Virus Research (Institute for Frontier Life and Medical Sciences since October in 2016), Kyoto University, Kyoto, Japan.

## Author Contributions

ND and TM designed the research, performed the experiments, and discussed the results. HM and HS performed the experiments, and discussed the results. TK discussed the results. AA designed the research, discussed the results, and wrote the manuscript. MN designed the research, performed the experiments, discussed the results, and wrote the manuscript. All authors approved its submission.

## Conflict of Interest Statement

The authors declare that the research was conducted in the absence of any commercial or financial relationships that could be construed as a potential conflict of interest.
